# Design of a Compact Quad-Channel Microstrip Diplexer for L and S Band Applications

**DOI:** 10.3390/mi14030553

**Published:** 2023-02-26

**Authors:** Sobhan Roshani, Salah I. Yahya, Yaqeen Sabah Mezaal, Muhammad Akmal Chaudhary, Aqeel A. Al-Hilali, Afshin Mojirleilani, Saeed Roshani

**Affiliations:** 1 Department of Electrical Engineering, Kermanshah Branch, Islamic Azad University, Kermanshah 6718997551, Iran; 2 Department of Communication and Computer Engineering, Cihan University-Erbil, Erbil 44001, Iraq; 3 Department of Software Engineering, Faculty of Engineering, Koya University, Koya KOY45, Iraq; 4 Medical Instrumentation Engineering Department, Al-Esraa University College, Baghdad 10011, Iraq; 5 Department of Electrical and Computer Engineering, College of Engineering and Information Technology, Ajman University, Ajman 346, United Arab Emirates; 6 College of Medical Techniques, Al-Farahidi University, Baghdad 10011, Iraq

**Keywords:** bandpass filter, diplexer, quad-channel, resonator, coupled lines, microstrip, insertion loss, return loss

## Abstract

In this paper, two novel dual-band bandpass filters (BPFs) and a compact quad-channel diplexer working at 1.7/3.3 GHz and 1.9/3.6 GHz are proposed. In the proposed diplexer design, triangular loop resonators and rectangular loop resonators are used together to reduce the circuit size and improve diplexer performances. Insertion loss (IL) and return loss (RL) of the proposed diplexer are better than 0.8 dB and 21 dB, respectively, at these four operating frequencies. Output ports isolation parameter is better than 30 dB. With the achieved specifications, the proposed diplexer can be used in L and S band applications.

## 1. Introduction

Diplexers are three-port devices, which are widely used in microwave circuits and systems. The diplexer devices split input signal from the common input port into two separate channels with two different desirable operating frequencies [1,2]. Microstrip diplexers are considered as key component in many communication systems. In many applications, diplexers allow a single antenna to receive and transmit on different frequencies. Moreover, diplexers will provide the ability for an antenna to receive and transmit simultaneously [3]. In recent reported works, hairpin resonators [4], stepped-impedance resonators [5], bandpass filters (BPFs) [6], square ring resonators [7] approaches are used to design and improve the performance of the diplexers. In [4], two hairpin line resonators are used in the diplexer structure to obtain two wide operating bands. Five stepped-impedance resonators are used to achieve a diplexer with compact size and high isolation in [5].

Recently, optimization algorithms [8,9] and neural network techniques have been used to improve performance of electronic circuits, such as in [10,11,12,13,14], which also have been used in the designing of the BPF [15] and coupler [16]. In [15], a narrow band BPF at 2.2 GHz is designed, with a hairpin structure. An artificial neural network (ANN) is used to optimized BPF, and in [16], an ANN model is used to find transfer function of the branch line coupler. Additionally, higher frequencies for filters and resonators have been achieved using optical fiber substrates [17,18,19,20,21,22].

Additionally, lumped reactive components such as capacitors and inductors are used in microwave circuits to provide a bandpass response, such as in [23,24,25]. Applied lumped reactive components increase insertion loss, which is not desirable.

Different kinds of resonators are also used for the performance improvement of the frequency response [26,27,28,29,30,31,32,33,34,35,36]. Different shapes of the resonators have been recently presented, such as U-shaped [26], T-shaped [27], Pi-shaped, [28] stepped-impedance [29], and patch resonators [30,31]. In [30], patch resonators are used to have a filtering response.

This paper presents a compact diplexer formed by two dual-band bandpass filters using triangular loop resonators and rectangular loop resonators operating at 1.7/3.3 GHz and 1.9/3.6 GHz. The proposed quad-channel diplexer is designed for L band and S band applications, which includes 1–2 GHz for L band and 2–4 GHz for S band.

## 2. Bandpass Filters Design

**Figure 1:**
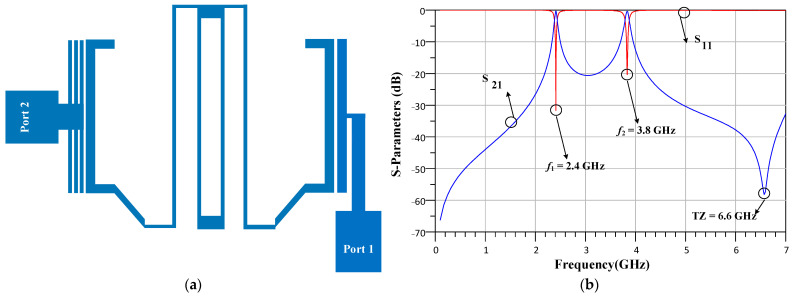
The (**a**) structure and (**b**) response of resonator1.

The proposed diplexer consists of two dual-band BPFs. At the first step of design process, the BPFs structure are introduced. The BPFS are designed using triangular loop and rectangular loop resonators to form a microstrip quad-channel diplexer for L band and S band applications. At the first step, coupled lines and rectangular loop resonators are combined to provide a dual-band resonator, named resonator1. The structure and response of resonator1 are depicted in Figure 1a,b. As seen, resonator1 provides two narrow operating bands at 2.4 GHz and 3.8 GHz. Additionally, the resonator1 creates a transmission zero (TZ) at 6.6 GHz, which provides a stop band near this transmission zero.

**Figure 2:**
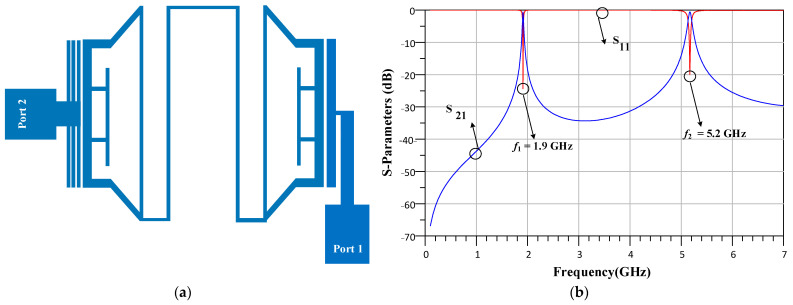
The (**a**) structure and (**b**) response of resonator2.

At the second step, triangular loop resonators and Pi-shaped resonators are incorporated to form resonator2. The structure and response of resonator2 are depicted in Figure 2a,b. Resonator2 provides two operating bands at 1.9 GHz and 5.2 GHz. As seen, resonator2 cannot provide a stopband with high attenuation level.

**Figure 3:**
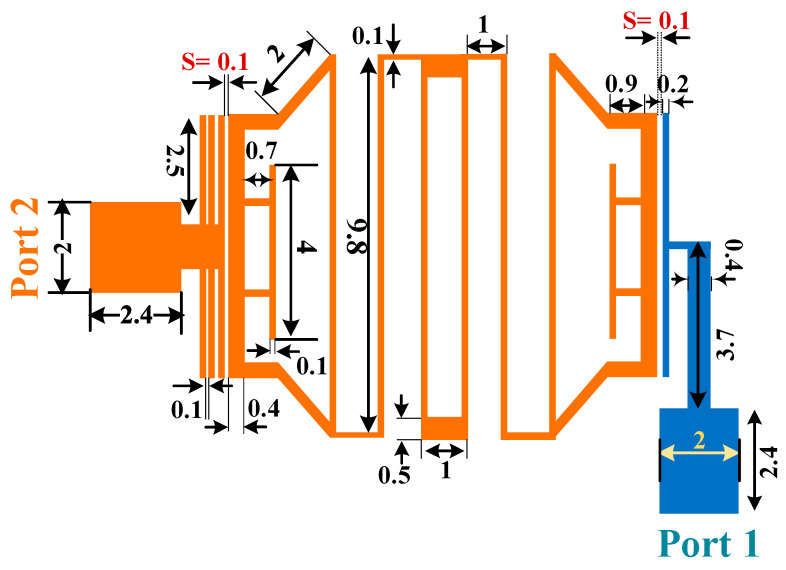
Structure of the first proposed BPF at 1.9/3.6 GHz.

**Figure 4:**
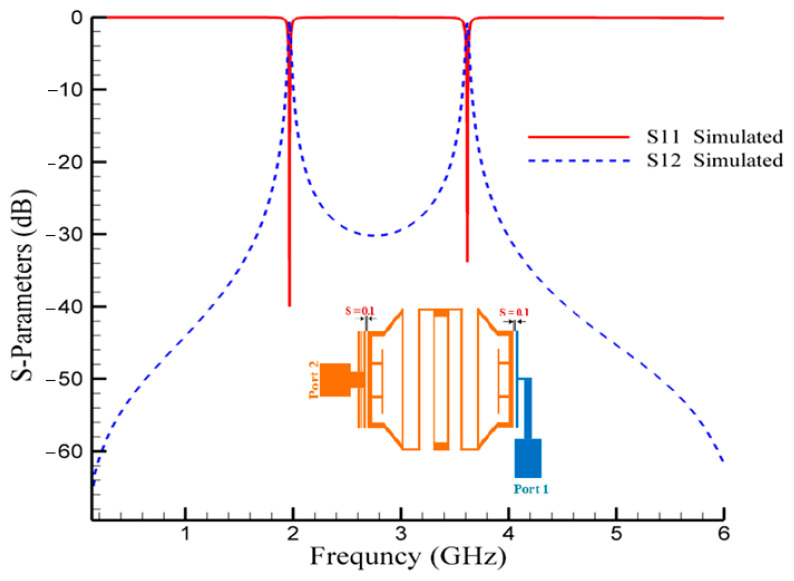
Simulated frequency responses of the first proposed BPF at 1.9/3.6 GHz.

In order to create a compact BPF with high attenuated stop band, resonator1 and resonator2 are combined to form the final structure of the first BPF. Figure 3 shows the structure of the first designed band-pass filter, which passes signals at 1.9 GHz and 3.6 GHz frequencies and suppresses other frequencies. The simulated frequency responses of this proposed filter are depicted in Figure 4. The insertion losses (IL) at the operating frequencies are 0.52 dB and 0.76 dB, and the return losses (RL) parameter values are better than 40 dB and 33 dB, respectively.

**Figure 5:**
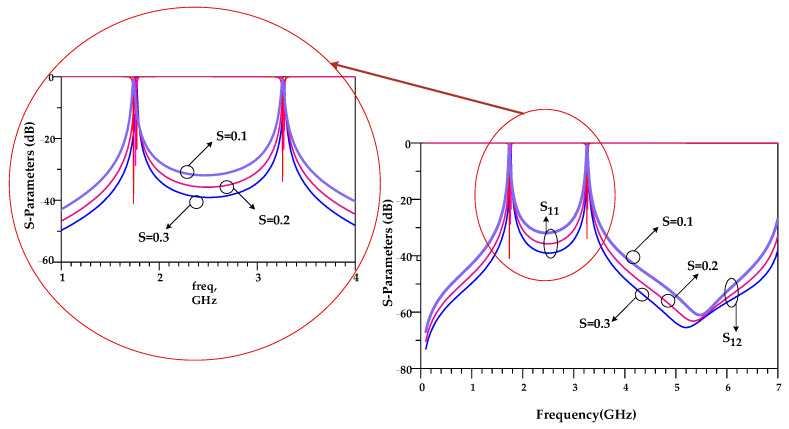
Effects of gap space (S) in the first proposed BPF at 1.9/3.6 GHz.

In the structure of the proposed BPF shown in Figure 3, there are two space gaps, which creates coupling. These gaps, which are demonstrated with “S”, are very important. As seen in Figure 5, by tuning the values of S, the operating frequency and the IL can be adjusted. The lowest insertion loss is obtained for S = 0.1 mm.

**Figure 6:**
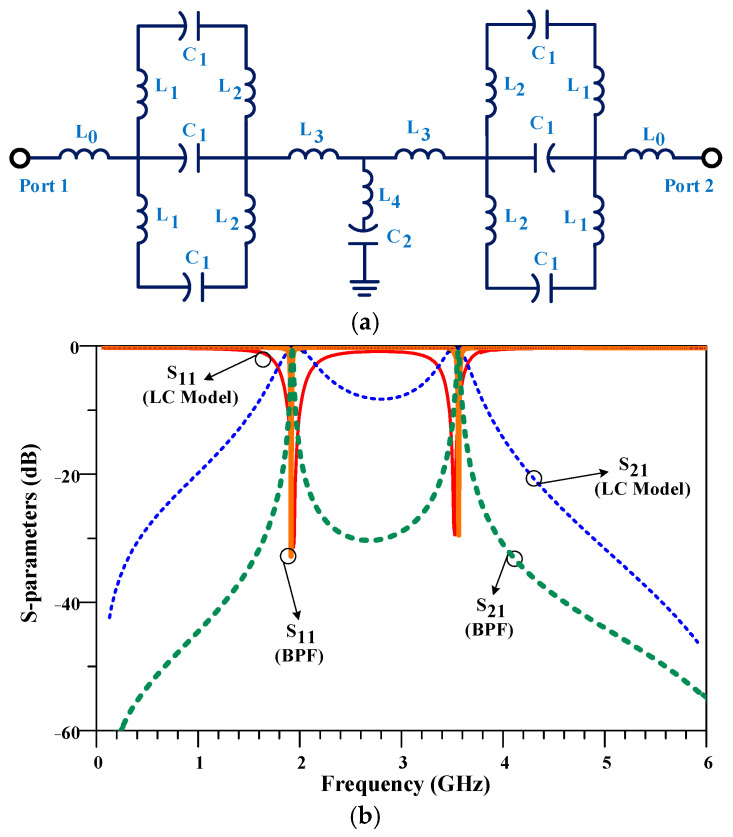
The (**a**) schematic of simplified LC equivalent circuit model and (**b**) its frequency response for the first proposed BPF at 1.9/3.6 GHz. The circuit parameters of the proposed LC model are as follows: L_0_ = 5 nH, L_1_ = 8.2 nH, L_2_ = 8.5 nH, L_3_ = 13.3 nH, L_4_ = 2.1 nH, C_1_ = 0.1 pF, and C_2_ = 0.19 pF.

The simplified LC equivalent circuit for the first proposed BPF at 1.9/3.6 GHz is illustrated in Figure 6a. Additionally, the frequency response of the LC model and the proposed BPF are compared in Figure 6b, which shows good agreement between the obtained S-parameters.

**Figure 7:**
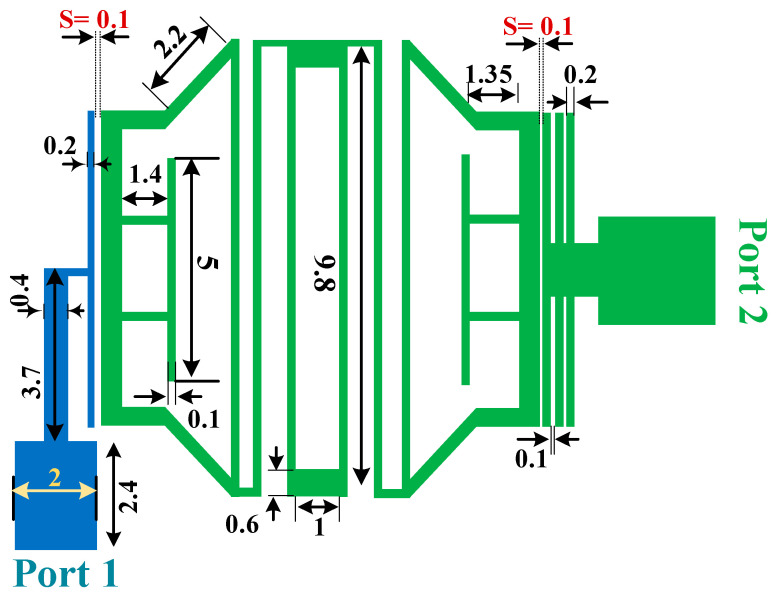
Structure of the second proposed BPF at 1.7/3.3 GHz.

**Figure 8:**
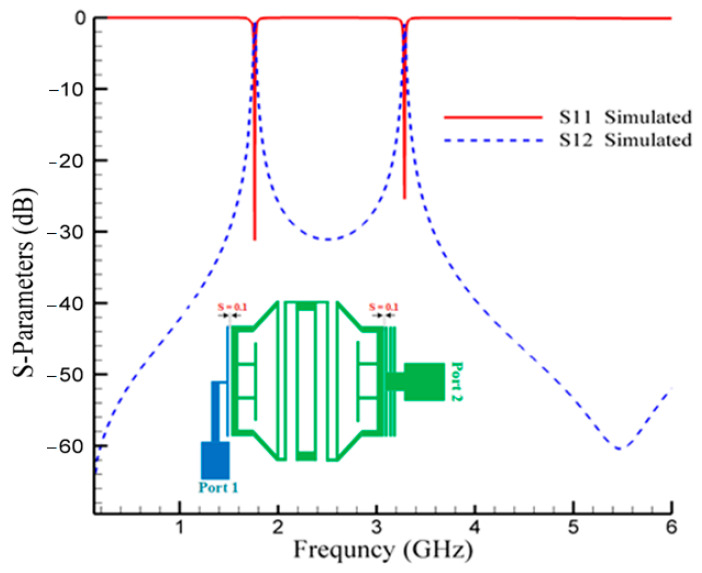
Simulated frequency responses of the second proposed BPF at 1.7/3.3 GHz.

Figure 7 shows the structure of the second designed BPF, which passes signals at 1.7 GHz and 3.3 GHz frequencies and suppresses other frequencies. The simulated frequency responses of this proposed filter are depicted in Figure 8. The ILs at operating frequencies are 0.53 and 0.86 dB, and the RLs parameter are better than 32 dB and 25 dB, respectively.

**Figure 9:**
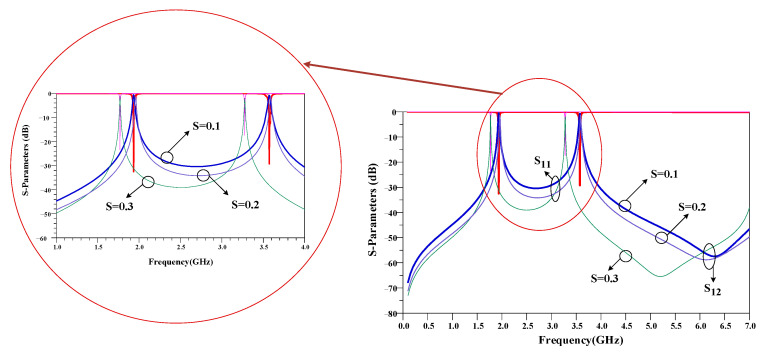
Effects of gap space (S) in the second proposed BPF at 1.7/3.3 GHz.

Like the first BPF, in the structure of the proposed second BPF, as seen in Figure 7, there are two space gaps, which creates coupling. These gaps, which are demonstrated with “S”, are very important. As seen in Figure 9, by tuning the values of S, the operating frequency and the IL can be adjusted. The lowest insertion loss is obtained for S = 0.1 mm.

**Figure 10:**
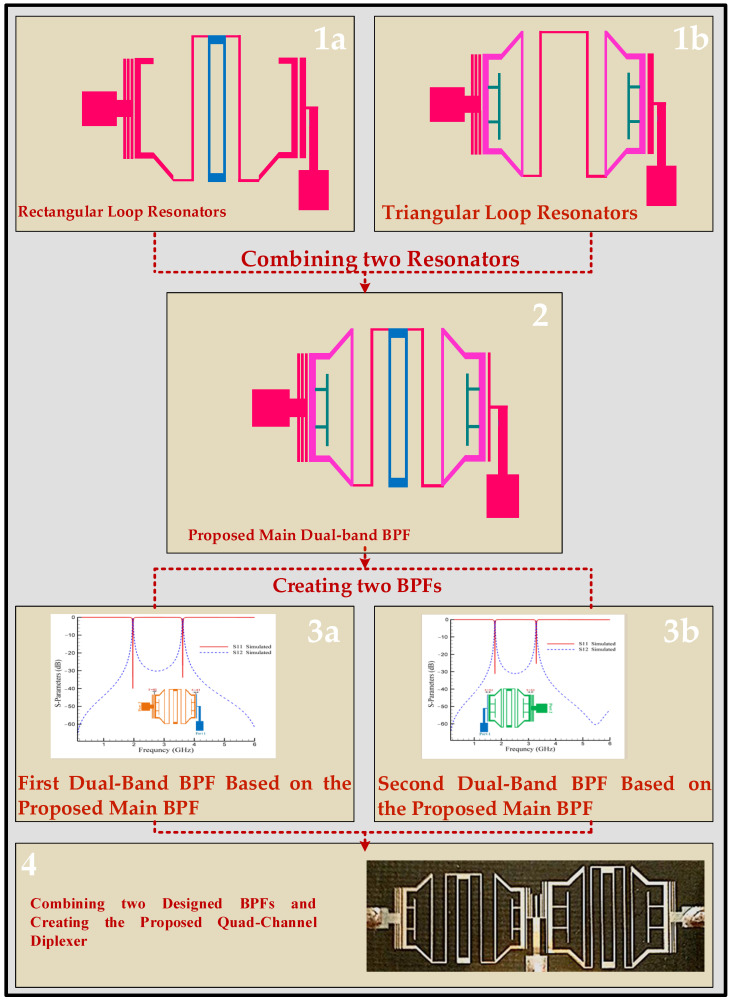
Design procedure of the proposed diplexer. The design steps of the proposed quad-channel diplexer are explained in four steps, which are indicated in the figure.

The design procedure of the proposed diplexer is depicted in Figure 10. In step1, rectangular loop and triangular loop resonators are designed. In step2, the designed rectangular loop and triangular loop resonators are combined to form the main dual-band proposed BPF. Then, based on the proposed main dual-band BPF, two BPFs are presented to provide four channels for the diplexer, as shown in step3. Additionally, in step4, the proposed quad-channel diplexer is presented by combining the two designed BPFs.

## 3. Diplexer Design

**Figure 11:**
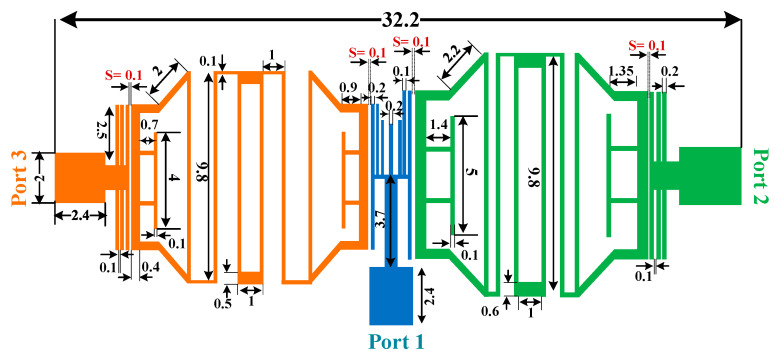
Proposed diplexer layout with dimensions (all in mm).

The proposed diplexer consists of two dual-band BPFs and a T-junction connection at input port. Each filter is constructed using the coupled stepped-impedance resonators (CSIRs), and two type of resonators, triangular loop resonators and rectangular loop resonators. The layout of the proposed diplexer is depicted in Figure 11. All the dimensions shown in this figure are in mm. By using the coupled open stubs in the diplexer structure, the parameters of insertion loss, isolation and stopbands are improved slightly. Additionally, one of the three coupled open stubs, which is closer to the BPF, creates the main coupling between the BPFs and diplexer ports.

**Figure 12:**
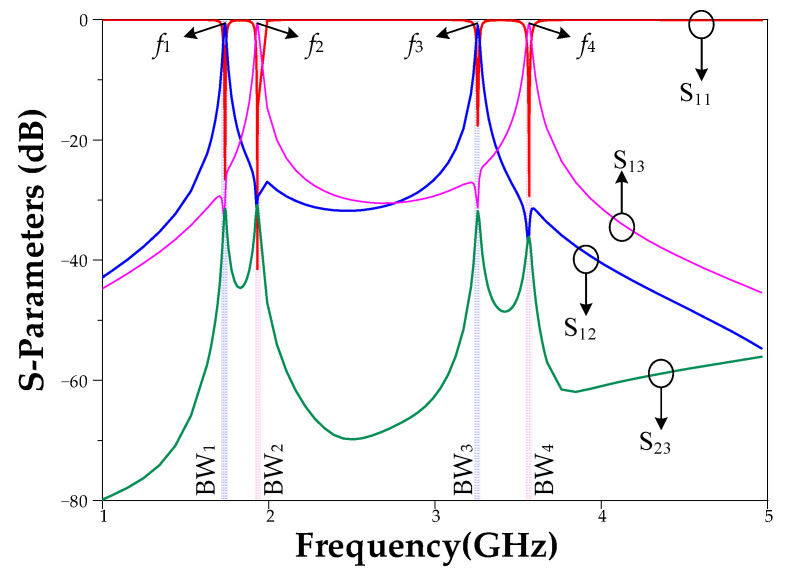
The S-parameter of the proposed diplexer with four center frequencies of 1700 MHz, 1900 MHz, 3300 MHz, and 3600 MHz.

The simulation S-parameter result of the proposed diplexer is shown in Figure 12. Four operating frequencies with 30 MHz bandwidths are achieved for the designed diplexer. Additionally, the isolation values for all operating bandwidths are below 30 dB, which is a desirable parameter for the designed diplexer. The four operating bands are 1685–1715 MHz with a center of 1700 MHz, 1885–1915 MHz with a center of 1900 MHz, 3285–3315 MHz with a center of 3300 MHz, and 3585–3615 MHz with a center of 3600 MHz.

## 4. Results and Discussion

**Figure 13:**
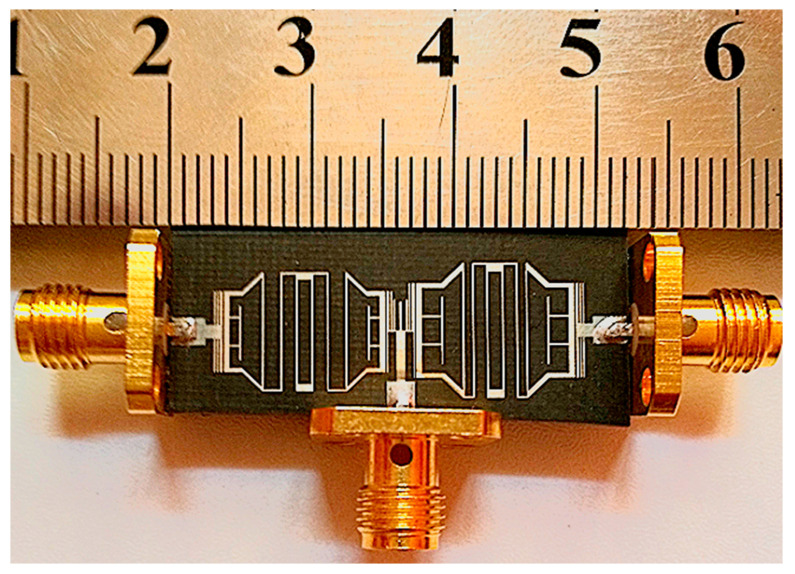
Fabricated photo of the proposed diplexer.

The final dimensions of diplexer are only 11.2 mm × 32.2 mm (0.0903 λg × 0.259 λg). Figure 13 shows the photograph of the fabricated diplexer. The proposed quad-channel diplexer, which is working at 1.7/3.3 GHz and 1.9/3.6 GHz, is designed and fabricated on a single layer of RT Duroid 5880 substrate with a relative electric constant of ε_r_ = 2.2, tanδ = 0.0009, and thickness of 0.7874 mm.

**Figure 14:**
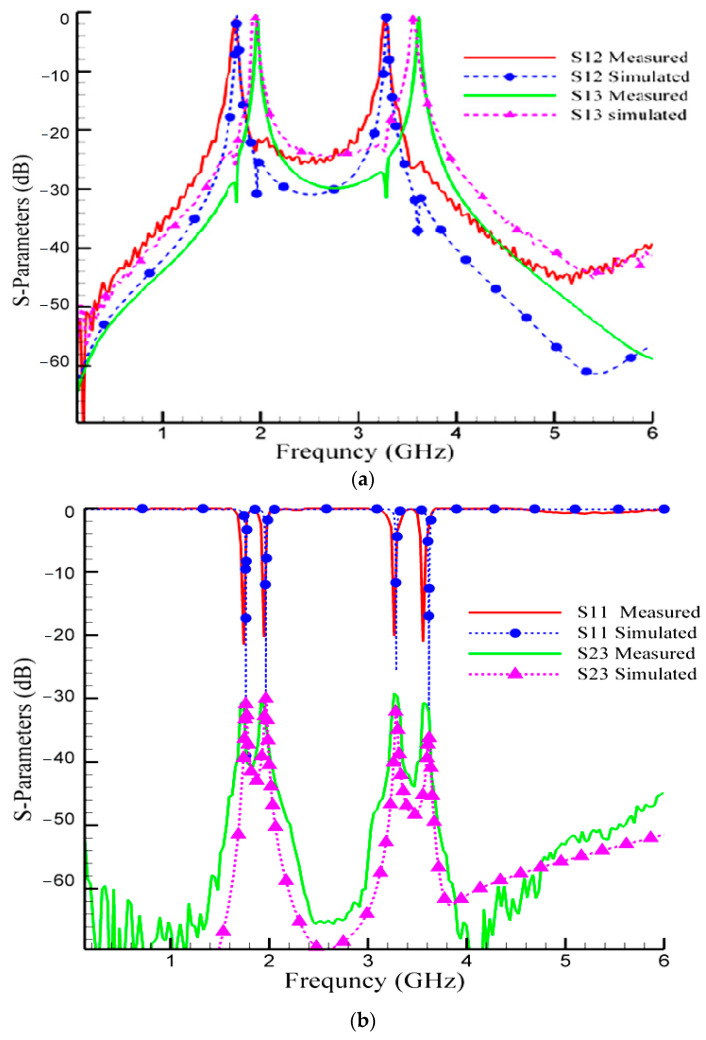
Simulation and measurement results of the proposed diplexer (**a**) scattering parameters (S_12_,S_13_) and (**b**) isolation parameter and input return loss (S_11_,S_23_).

Port one represents the input port connected to the antenna, whereas port two and port three represent the output of the receiver filter and the input of the transmitter filter, respectively. All ports are designed for 50 Ohms impedance. Figure 14a,b shows the simulation and measurement results of the proposed diplexer. As seen in these figures, the proposed diplexer has two channels. The lower channel has two frequency bands 1.7/1.9 GHz, whereas the higher channel has two frequency bands 3.3/3.6 GHz. According to the fabrication measured results, the insertion loss parameters of the proposed diplexer are better than 0.6 dB at the lower channel and better than 0.8 dB at the higher channel. The measured return loss parameters are better than 20 dB and 25 dB at the lower and higher channel, respectively. Moreover, better than 30 dB ports isolation is obtained in the whole frequency band.

The simulated results of the proposed diplexer are listed in Table 1. As the results show, the proposed diplexer features very good specifications. In the lower bands (1.7 GHz and 1.9 GHz), the S_21_ parameter at 1.7 GHz is achieved (−0.55 dB), while the S_31_ parameter at 1.9 GHz is achieved (−0.55 dB); therefore, the insertion loss in lower bands is 0.55 dB. In the higher bands (3.3 GHz and 3.6 GHz), the S_21_ parameter at 3.3 GHz is achieved (−0.87 dB), while the S_31_ parameter at 3.6 GHz is achieved (−0.78 dB); therefore, the insertion loss in higher bands is better than 0.87 dB.

In the lower bands (1.7 GHz and 1.9 GHz), the S_11_ parameter for these two frequencies is achieved (−23.3 dB and −21.1 dB, respectively); therefore, the return loss in lower bands is better than 21 dB. In the higher bands (3.3 GHz and 3.6 GHz), the S_11_ parameter for these two frequencies is achieved (−25.64 dB and −25.67 dB, respectively); therefore, the return loss in higher bands is better than 25 dB.

In the lower bands (1.7 GHz and 1.9 GHz), the S_23_ parameter for these two frequencies is achieved (−30.83 dB and −30.04 dB, respectively); therefore, the isolation in lower bands is better 30 dB. In the higher bands (3.3 GHz and 3.6 GHz), the S_23_ parameter for these two frequencies is achieved (−31.32 dB and −36.1 dB, respectively); therefore, the isolation in higher bands is better than 31 dB.

**Figure 15:**
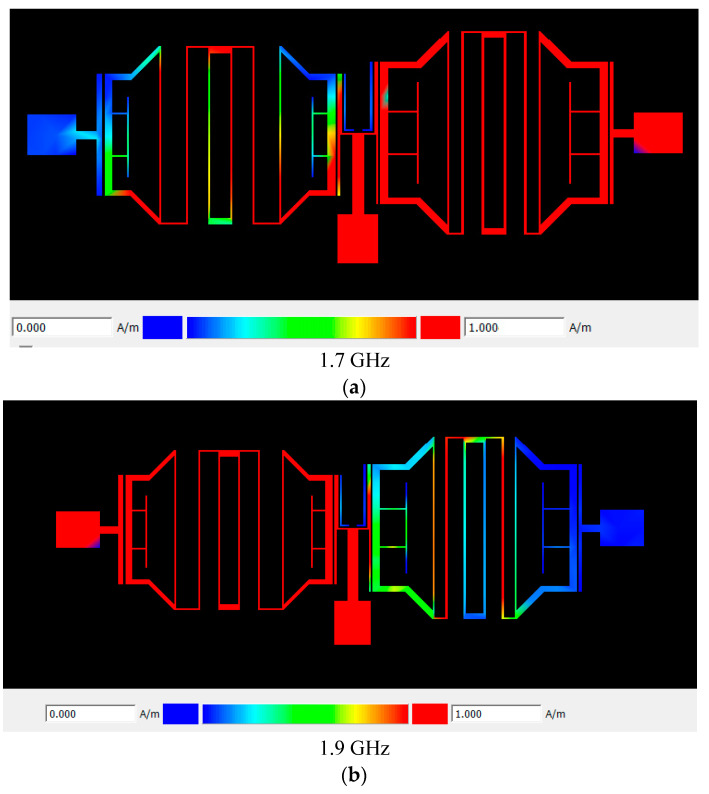

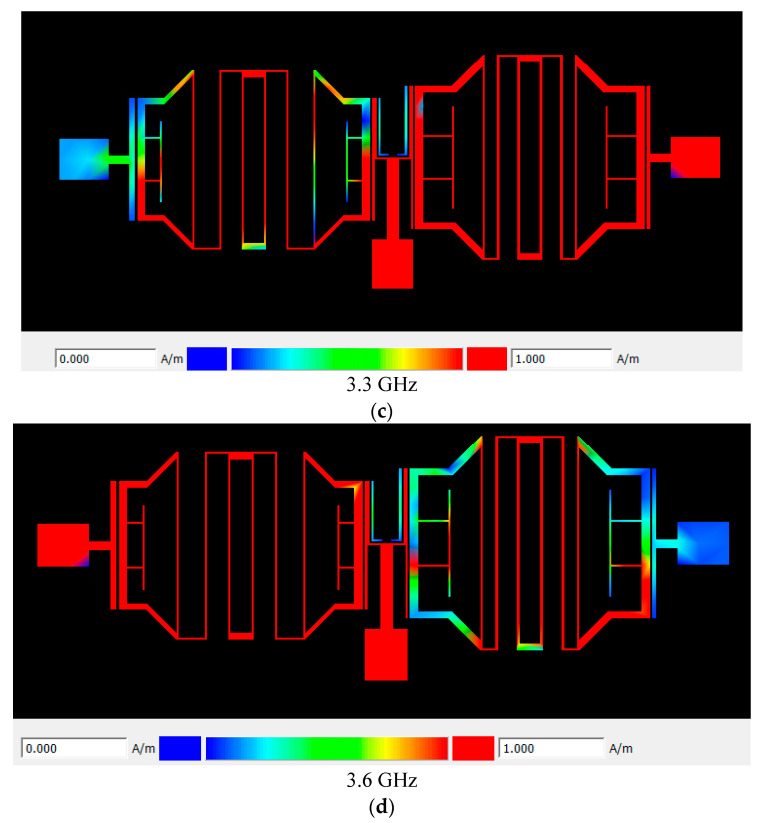
Surface current distribution in the proposed diplexer at the frequencies of: (**a**) 1.7 GHz, first frequency band in port2; (**b**) 1.9 GHz, second frequency band in port3; (**c**) 3.3 GHz, third frequency band in port2; and (**d**) 3.6 GHz, fourth frequency band in port3. The maximum value of magnetic intensity is 1 A/M in all of cases.

The surface current distributions in the proposed quad-band diplexer are demonstrated in Figure 15a–d. The proposed diplexer correctly works at four frequency bands of 1.7/1.9/3.3/3.6 GHz. As per the results shown in Figure 15a,c, the currents are correctly distributed uniformly at the port2 at the 1.7 GHz and 3.3 GHz frequencies and show that the currents have not reached the port3. Additionally, as seen in Figure 15b,d, the results show that the currents are correctly distributed uniformly at the port3 at the 1.9 GHz and 3.6 GHz frequencies and show that the currents have not reached the port2.

The proposed diplexer has good features, where the S-parameters of the proposed device at the four operating frequencies are listed in Table 2.

A performance comparison between the designed diplexer with the previous reported diplexers is listed in Table 3. As seen in this table, most of the reported works focus on a dual-band diplexer, but the proposed diplexer operates at four frequencies. The proposed quad-channel diplexer shows good performance, compared to the reported works. The designed diplexer has the smallest size and lowest ILs, as compared with other reported works.

## 5. Conclusions

In this paper, a compact quad-channel diplexer is designed, simulated and fabricated. The proposed structure is composed of two BPFs. In the proposed design, triangular loop and rectangular loop resonators are used together in order to reduce the circuit size and optimize the specifications of the proposed circuit. The proposed diplexer operates correctly at 1.7 GHz, 1.9 GHz, 3.3 GHz, and 3.6 GHz frequencies. The measured ILs are better than 0.8 dB, and the RLs are better than 20 dB at the four operating frequencies. Moreover, better than 30 dB ports isolation is obtained in the whole frequency band. With these specifications, the proposed diplexer can be useful for L band and S band applications.

## Figures and Tables

**Table 1:** Specifications of the proposed diplexer.

Parameter	Unit	Lower Bands	Higher Bands
Frequency	GHz	1.7/1.9	3.3/3.6
Insertion loss	dB	0.55	Better than 0.87
Return loss	dB	Better than 21	Better than 25
Isolation	dB	Better than 30	Better than 31

**Table 2:** Scattering parameters of the designed device.

S-Parameters (dB)	Frequency (GHz)			
	1.7	1.9	3.3	3.6
S_11_	−23.30	−21.1	−25.64	−25.67
S_12_	−0.55	−30.63	−0.87	−35.93
S_13_	−29.44	−0.55	−30.20	−0.78
S_23_	−30.83	−30.04	−31.32	−36.10

**Table 3:** Comparison between the designed devices with the previous diplexers.

Ref.	Lower Band (1) (dB)		Higher Band (1) (dB)		Lower Band (1) (dB)		Higher Band (1) (dB)		Lower Band (1) (dB)		Higher Band (1) (dB)		Lower Band (2) (GHz)		Higher Band (2) (GHz)		Size	
	*IL*_1_	*IL*_2_	*IL*_3_	*IL*_4_	*IRL*_1_	*IRL*_2_	*IRL*_3_	*IRL*_4_	*I*_1_	*I*_2_	*I*_3_	*I*_4_	*f*_1_	*f*_2_	*f*_3_	*f*_4_	mm^2^	λg^2^
This work	0.53	0.55	0.87	0.78	23	21	25	25	30	30	31	36	1.7	1.9	3.3	3.6	360.64	0.0233
[37]	0.8	1	0.7	1.5	24	21	23	22	50	30	45	30	1.5	2	2.4	3.5	1456	0.078
[38]	1.55	-	1.70	-	21	-	31	-	45	-	41	-	1.8	-	2.2	-	923.4	0.0667
[39]	2.2	-	2.1	-	27	-	26	-	30	-	30	-	1.82	-	2.41	-	859.32	0.0646
[40]	1.25	-	1.48	-	25	-	14	-	35	-	30	-	2.16	-	2.91	-	256	0.470
[41]	1.34	-	0.95	-	22	-	21	-	24	-	22	-	1.81	-	2.44	-	1040	0.179
[42]	2.1	-	2.1	-	20	-	20	-	20	-	20	-	1.75	-	1.85	-	918	0.0705
[43]	0.6	-	0.9	-	11	-	12	-	13	-	23	-	2.6	-	6	-	573.11	0.0809
[44]	1.5	-	1.3	-	21	-	21	-	31	-	35	-	2.34	-	2.59	-	816	0.1019

The parameter of ILi represents insertion loss, IRLi corresponds to input return loss, and Ii represents isolation.

## Data Availability

All the material conducted in the study is mentioned in article.
